# Mutation in KERA Identified by Linkage Analysis and Targeted Resequencing in a Pedigree with Premature Atherosclerosis

**DOI:** 10.1371/journal.pone.0098289

**Published:** 2014-05-30

**Authors:** Stephanie Maiwald, Suthesh Sivapalaratnam, Mahdi M. Motazacker, Julian C. van Capelleveen, Ilze Bot, Saskia C. de Jager, Miranda van Eck, Jennifer Jolley, Johan Kuiper, Jonathon Stephens, Cornelius A. Albers, C. Ruben Vosmeer, Heleen Kruize, Daan P. Geerke, Allard C. van der Wal, Chris M. van der Loos, John J. P. Kastelein, Mieke D. Trip, Willem H. Ouwehand, Geesje M. Dallinga-Thie, G. Kees Hovingh

**Affiliations:** 1 Department of Vascular Medicine, Academic Medical Centre, Amsterdam, the Netherlands; 2 Department of Experimental Vascular Medicine, Academic Medical Centre, Amsterdam, the Netherlands; 3 Division of Biopharmaceutics, Leiden/Amsterdam Centre for Drug Research, Leiden, the Netherlands; 4 Department of Haematology, University of Cambridge, Cambridge, United Kingdom; 5 National Health Service Blood and Transplant, Cambridge, United Kingdom; 6 Amsterdam Institute of Molecules, Medicines and Systems, Division of Molecular and Computational Toxicology, Department of Chemistry and Pharmaceutical Sciences, VU University, Amsterdam, the Netherlands; 7 Department of Pathology, Academic Medical Centre, Amsterdam, the Netherlands; 8 Department of Human Genetics, Wellcome Trust Sanger Institute, Hinxton, United Kingdom; Innsbruck Medical University, Austria

## Abstract

**Aims:**

Genetic factors explain a proportion of the inter-individual variation in the risk for atherosclerotic events, but the genetic basis of atherosclerosis and atherothrombosis in families with Mendelian forms of premature atherosclerosis is incompletely understood. We set out to unravel the molecular pathology in a large kindred with an autosomal dominant inherited form of premature atherosclerosis.

**Methods and Results:**

Parametric linkage analysis was performed in a pedigree comprising 4 generations, of which a total of 11 members suffered from premature vascular events. A parametric LOD-score of 3.31 was observed for a 4.4 Mb interval on chromosome 12. Upon sequencing, a non-synonymous variant in *KERA* (c.920C>G; p.Ser307Cys) was identified. The variant was absent from nearly 28,000 individuals, including 2,571 patients with premature atherosclerosis. KERA, a proteoglycan protein, was expressed in lipid-rich areas of human atherosclerotic lesions, but not in healthy arterial specimens. Moreover, KERA expression in plaques was significantly associated with plaque size in a carotid-collar *Apoe^−/−^* mice (r^2^ = 0.69; p<0.0001).

**Conclusion:**

A rare variant in *KERA* was identified in a large kindred with premature atherosclerosis. The identification of KERA in atherosclerotic plaque specimen in humans and mice lends support to its potential role in atherosclerosis.

## Introduction

In both cardiovascular disease (CVD) and stroke, atherosclerosis is the underlying pathology. Genetic factors explain a proportion of the observed inter-individual variability in atherosclerosis progression, which is exemplified by the observed 30–60% heritability in twin studies [1], and the finding that a positive family history for premature atherosclerosis is an independent risk factor [2]. Both common and rare genetic variants contribute to the heritability [3]. A recent meta-analysis of Genome Wide Association Studies (GWAS) of nearly 64,000 cases with CVD has identified 46 common single nucleotide polymorphisms (SNPs) of small effect size, which account for about 10.6% of the estimated heritability [4]. The remaining heritability is assumed to be explained by a combination of common variants with effect sizes so small that they remained undetected in the recent GWAS meta-analysis, by rare variants with an intermediate effect, and by pedigree-specific mutations with a large effect. The latter have been identified in several pedigrees with Mendelian forms of atherosclerosis [5]–[11]. A well-known example of such a monogenic dominant disorder, that underlies atherosclerosis, is Familial Hypercholesterolemia (FH), caused by loss of function (LOF) causing mutations in the genes encoding for the Low-density lipoprotein receptor (*LDLR*) or Apolipoprotein B (*APOB*), or gain of function (GOF) mutations in Proprotein convertase subtilisin/kexin type 9 (*PCSK9*). Carriers of mutations in these genes are characterized by high plasma levels of LDL-cholesterol (LDL-c) and early onset atherosclerosis [12].

The molecular basis of premature atherosclerosis in the absence of high LDL-c levels is largely unknown, but recently rare and putative causal variants in Myocyte enhancer factor–2 (*MEF2A*) and Low-density lipoprotein receptor-related protein 6 (*LRP6*) have been identified in pedigrees with Mendelian forms of atherosclerosis where FH as a causal factor was ruled out [5], [10], [11]. The predictive power of a family based approach has recently been documented by Erdmann and co-workers [13], who identified 2 novel private mutations in Guanylate Cyclase 1 soluble alpha 3 (*GUCY1A3)* and Chaperone Containing TCP1 subunit 7 (*CCT7)*, in a pedigree with a mendelian form of CVD. The identification of such mutations in novel genes provides new and pivotal information about the pathobiology of premature atherosclerosis, and may ultimately lead to new pharmacological interventions to address the burden of atherosclerosis.

The aim of the current study was to identify the molecular defect in a large non-FH pedigree with an autosomal dominant form of atherosclerosis. We identified a non-synonymous (ns) mutation in the Keratocan (*KERA*) gene, which encodes the extracellular proteoglycan KERA. Additional genetic, histological and animal studies were performed to further establish the role of this variant in atherosclerosis.

## Methods

### Recruitment of the Pedigree with Early Onset Atherosclerosis

**Figure 1:**
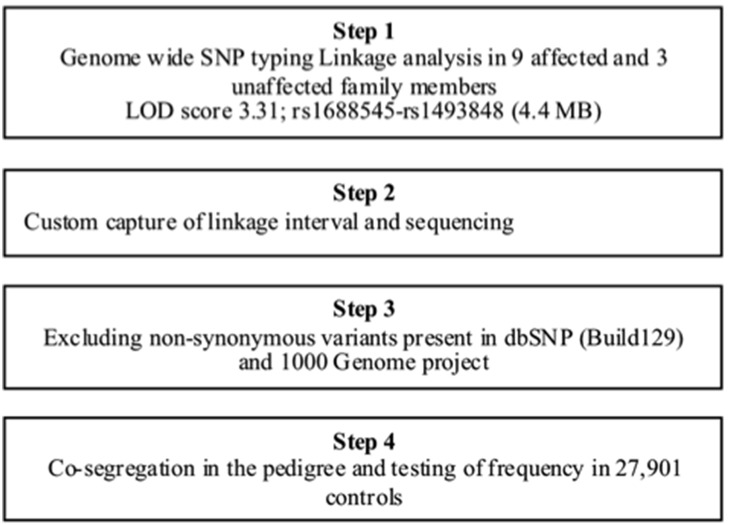
Schematic overview of the gene discovery strategy.

A male subject who suffered from an acute myocardial infarction (AMI) at the age of 49 years was referred to the outpatient clinic of the Academic Medical Center (AMC) in Amsterdam (Figure 1B; index case III:8). An autosomal dominant form of inheritance of early onset atherosclerosis in the pedigree was identified. Premature atherosclerosis was defined as a documented atherosclerotic event, either CVD or stroke, before the age of 55 (male) and 65 (female). Blood was obtained after an overnight fast. Plasma was isolated by centrifugation at 3000 rpm, for 20 minutes at 4°C and was stored at −80°C for further analyses. Plasma cholesterol, LDL-c, high-density lipoprotein cholesterol (HDL-c) and triglycerides (TG) were analysed using commercially available assays (Randox, Antrim, United Kingdom and Wako, Neuss, Germany) on a Cobas-Mira autoanalyzer (Roche, Basel, Switzerland). Hypertension was defined as a systolic blood pressure >140 mmHg and/or diastolic blood pressure >90 mmHg or the use of anti-hypertensive lowering drugs. Diabetes mellitus was defined as fasting plasma glucose ≥7.0 mmol/l or use of anti-diabetic medication. The study complies with the Declaration of Helsinki and the Institutional Review Board of the AMC (Medische Ethische Toetsings Commissie, METC) of the University of Amsterdam approved the study. All participants provided written informed consent.

### Genetic Studies (Figure 2)

Genomic DNA was extracted from whole blood on an AutopureLS apparatus according to the manufacturer’s protocol (Gentra Systems, Minneapolis, MN, USA). Human CytoSNP-12 BeadChip kits were used for genome wide single nucleotide polymorphism (SNP) genotyping (Illumina, San Diego, CA, USA) in 12 relatives (Figure 1B; 9 affected and 3 unaffected). A Nimblegen (Madison, WI, USA) custom sequence capture array comprising 395K probes was designed to enrich for the genomic region that was identified by linkage analysis and used to sequence the DNA region with an Illumina GAII platform. Confirmation of the identified mutations and analysis of co-segregation of the variant in the pedigree was by Sanger sequencing as previously described [14]. The following primer pairs were used: *KERA*: exon 1 forward 5′-AAG ATT ACC AGC CAA TAC AAT GC-3′, reverse 5′-TGA TGG GAG ACC CTC ATC TG-3′ exon 2 forward: 5′-GCC ACT AAG CCC TCC ATA GG-3′; reverse (2) 5′-AGC AAT GGG GAA TAT GAC TTG-3′. After establishing the segregation in the core pedigree, the family was further expanded (Figure S1 in File S1).

**Figure 2:**
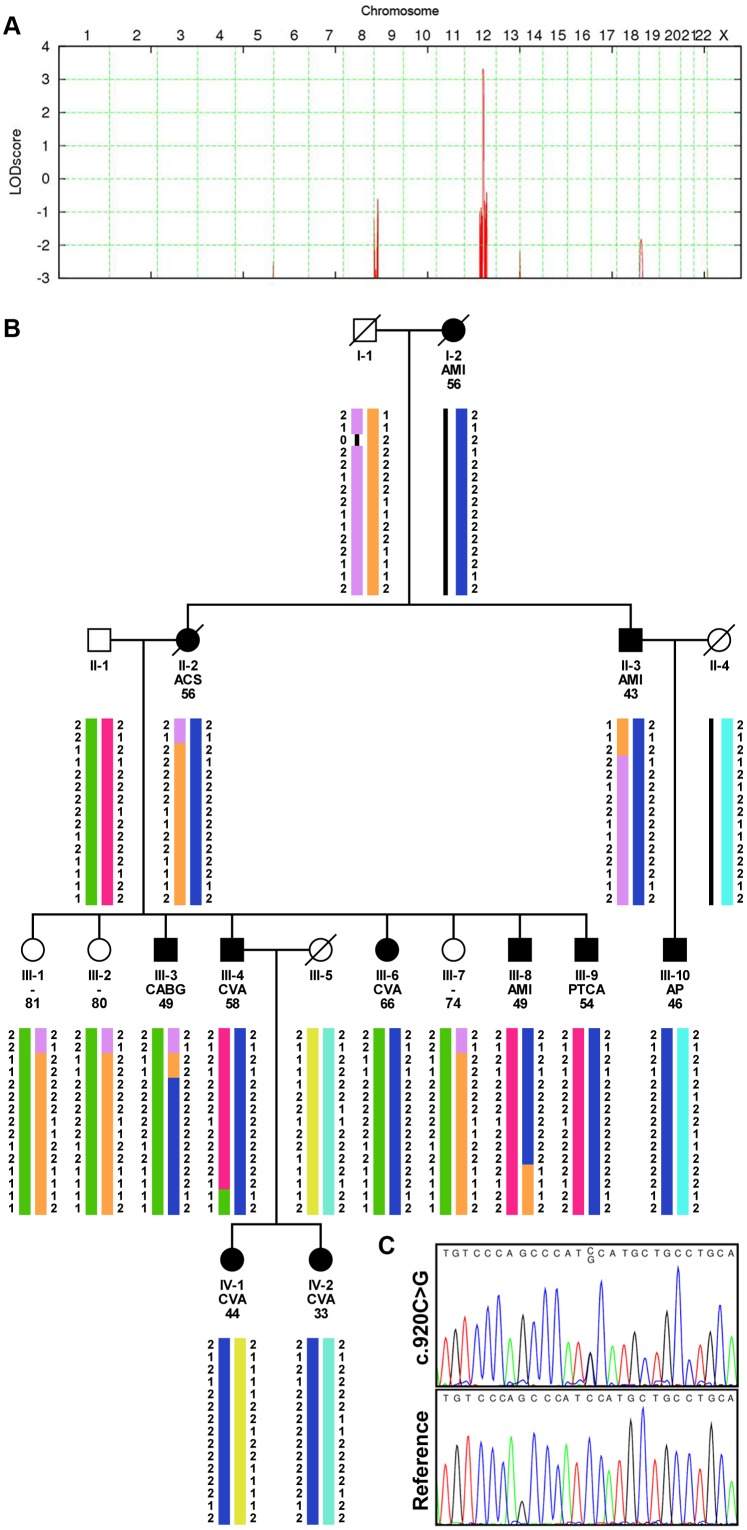
The identification of a gain of function mutation in *KERA*. **A**, A significant LOD-score of 3.31 was obtained on chromosome 12 after parametric linkage analysis by Allegro software using genotypes from 12 family members. **B**, Haplotype analyses of the linkage interval showing two recombination events in III-3 and III-8; top to bottom SNP order: rs11104542, rs7974491, rs10506959, rs1688545, rs1347846, rs704106, rs1948839, rs704144, rs790455, rs7980716, rs10777477, rs1493848, rs12308959, rs3847810, rs12426730. The linkage interval is defined by rs1688545 and rs1493848 and harbours 21 genes (Tables S2 and S3) Additional sequencing of the region led to the identification of the KERA p.Ser307Cys variant. This was the only rare non-synonymous variant. All affected relatives in this pedigree are heterozygous carriers of the *KERA* mutation. In the pedigree we show the type of event and the age at which the event occurred for each relative. III-8 is the index case. AMI = acute myocardial infarction; TIA = transient ischemic attack; PTCA = percutaneous transluminal coronary angioplasty; CVA = cerebrovascular accident; AP = angina pectoris; ACS = acute coronary syndrome; CABG = coronary artery bypass graft; **C**, DNA Sanger sequencing chromatogram showing the heterozygote c.920C>G; p.Ser307Cys *KERA* mutation.

### Validation Cohorts

The *KERA* variant was genotyped in:

1. Premature Atherosclerosis (PAS) Cohort: this cohort comprises 935 patients with early symptomatic atherosclerosis before the age of 51 years. Atherosclerosis is defined as myocardial infarction, coronary revascularization, or evidence of at least 70% stenosis in a major epicardial artery. [15] Patients were recruited at the cardiology and vascular outpatient clinic of the AMC. [16] To identify possible further cases with mutation or rare variants in the coding fraction of *KERA* the DNA samples of 296 randomly chosen PAS cases were sequenced.
2. Sanquin Blood Bank common Controls: DNA samples from 1,440 healthy volunteers were recruited from a large cohort of healthy blood donors, who were free of clinical CVD, at one of the collection clinics of the Sanquin Blood Bank covering the northwest section of the Netherlands, which geographically overlaps the PAS case cohort [16].
3. Cambridge BioResource Collection: DNA samples of 8,946 healthy volunteers were enrolled by NHS Blood and Transplant Unit in a resource for genotype-phenotype association studies [17]. In addition, genotyping results from 16,515 samples were retrieved from the UK10K (http://www.uk10k.org) and the NHLBI Exome Sequencing (ESP) projects [18].

### Human Plaque Quantification

Specimens of cornea (n = 2), tonsil (n = 2), mammary artery (n = 2), atherosclerotic- and non-diseased arteries (n = 9) were collected from patients at autopsy. The tissues from autopsy material for histological verification of protein expression were obtained from the Department of Pathology (Prof Dr AC van der Wal) within the AMC, Amsterdam. They were anonymously provided to us according to the GPC guidelines. No METC conformation was required. For details of the staining procedures see Supplemental Methods in File S1.

### Molecular Dynamics Computer Simulations

Molecular dynamics (MD) computer stimulations were performed to assess the effect of the p.Ser307Cys mutation on the structure of the KERA protein (see Supplemental Methods in File S1).

### Animal Experiments

The animal protocol was approved by the Ethics Committee for animal Experiments of the Leiden University (Leiden, The Netherlands) and carried out in compliance with the Dutch government guidelines. The animals were bred in house in the Gorleaus Laboratories of the Leiden/Amsterdam Center for Drug Research Leiden, the Netherlands. All surgery was performed under sodium pentobarbital anesthesia, and all efforts were made to minimize suffering. Male *Apoe^−/−^* mice, aged 10–12 weeks, were fed a Western type diet containing 0.25% cholesterol and 15% cacao butter (Special Diet Services, Sussex, UK) starting two weeks prior to collar placement surgery. Mice (n = 3–4 per group) were anaesthetized at t = 0 or 2, 4, 6, 8 and 10 weeks after collar placement and in-situ perfusion-fixation was performed, after which carotid arteries were sectioned and lesions were analyzed. [19] Morphometric analysis using Leica Qwin image analysis software (Leica Microsystems, Rijswijk, The Netherlands) was performed on hematoxylin and eosin stained 5 µm sections of the carotid arteries at the site of maximal stenosis as previously described. [19] Cryosections were stained for KERA protein using a rabbit anti KERA polyclonal antibody (clone H-50, Sc-66941) (Supplemental Methods in File S1).

### Statistical Analysis

Results are expressed as mean ± standard deviation unless otherwise stated. Two-sided P values of <0.05 were considered statistically significant. All statistical analysis were performed with SPSS version 18.0. Multipoint parametric linkage analysis assuming a fully penetrant autosomal dominant model, based on the clinical segregation of the disease phenotype in the pedigree and minor allele frequency (MAF) of <0.001 was conducted using Allegro software [20]. The identified variants were compared with the dbSNP (build 129) database and available data from the 1000 Genomes pilot project [21]. Only those variants that were not present in the database were selected for further testing.

## Results

### Identification of the Mutation in KERA

The index case III.8 (Figure 1B) suffered from an AMI at the age of 49 years, and apart from obesity, no other atherosclerosis risk factors were identified (Tables 1 and 2). Ten relatives did suffer from premature atherosclerosis, of whom 2 died prior to the conduct of this study. In addition to the nine living affected cases, we identified three unaffected relatives, who were above the predefined age for PAS and had not suffered from an atherosclerotic event. (Figure 1B, Tables 1 and 2). Genotyping was performed in these 12 individuals and multi-point linkage analysis resulted in a parametric LOD-score of 3.31 in a region located on chromosome 12q21.33-q22 (Figure 1A). The interval of 4.4 Mb was located between SNPs rs1688545 and rs1493848 (Figure 1B) and harboured 21 annotated genes (Table S1 in File S1 and Table S2 in File S1). Sequencing of the interval in the index case III.8 revealed 1 non-synonymous variant, which was absent from the 1000 Genomes project. This novel variant in exon 3 (NM_007035.3: c.920C>G; p.Ser307Cys, Figure 1C) was identified in a single *KERA* haplotype, which showed complete co-segregation with premature atherosclerosis in the core pedigree. Further expansion of the pedigree led to the identification of 10 additional *KERA* mutation carriers of whom 1 brother, aged 73 years, had no signs of atherosclerotic disease. Two female carriers of the mutation are still under the PAS age (Figure S1in File S1 and Table S3 in File S1). The *KERA* p.Ser307Cys variant was absent from 27,901 other DNA samples, including samples from 935 cases from the PAS cohort (Table S4 in File S1) and 1,636 cases of premature MI collection of the NHLBI Exome Sequencing project. The coding sequence and intron-exon boundaries of *KERA* were additionally sequenced in 296 PAS subjects and no additional rare variants with MAFs<0.5% were identified.

**Table 1:** Clinical characteristics of the core pedigree included in linkage analysis.

Patient	Sex	Type of CVD	Age CVD	Medication	KERA c.920C>G	Diabetes mellitus(years)	Hypertension(years)	Smoking	BMI (kg/m^2^)
III:1	F	None	N/A	None	No	No	No	Yes	20
III:2	F	None	N/A	None	No	No	Yes (55)	No	32
III:7	F	None	N/A	None	No	No	No	No	29
II:3	M	AMI	42	Simva 40 mg	Yes	No	No	No	32
III:3	M	CABG	49	Rosuva 10 mg	Yes	No	No	Yes	28
III:4	M	CVA	58	Prava 40 mg	Yes	Yes (54)	No	No	31
III:6	F	CVA	66	Atorva 20 mg	Yes	No	No	Yes	29
III:8†	M	AMI	49	Atorva 80 mg	Yes	No	No	No	35
III:9	M	PTCA	54	Simva 40 mg	Yes	No	No	Yes	30
III:10	M	AP	46	Rosuva 20 mg	Yes	No	No	No	38
IV:1	F	CVA	44	Atorva 20 mg	Yes	No	No	Yes	30
IV:2	F	CVA	33	Prava 20 mg	Yes	No	No	Yes	32

AMI = Acute Myocardial Infarction; AP = Angina Pectoris; PTCA = Percutaneous Transluminal Coronary Angioplasty; CABG = Coronary Artery Bypass Graft; CVA = Cerebrovascular Accident. Simva = Simvastatin, Rosuva = Rosuvastatin, Prava = Pravastatin and Atorva = Atorvastatin. BMI = body mass index (kg/cm^2^).

† = index case. Subjects were considered smokers if they were current smokers or when they quitted smoking within the last 5 years.

**Table 2:** Characteristics of the core pedigree included in linkage analysis.

	No atherosclerosis	Atherosclerosis present
Age	78±4	66±13
Sex (N = male/female) BMI	3/0 30.5±4.3	6/3 31.0±2.7
Total cholesterol (mmol/l)	6.4 [5.5–6.4]	4.1 [3.5–4.3]
LDL cholesterol (mmol/l)	4.2 [3.5–4.2]	2.1 [1.8–2.6]
HDL cholesterol (mmol/l)	1.7 [1.5–1.7]	1.1 [0.8–1.4]
Triglyceride (mmol/l)	1.2 [1.0–1.2]	1.3 [1.1–1.8]

Age is expressed as mean ± standard deviation and data are expressed as number (N). Lipid values are expressed as median with interquartile range (IQR). Pedigree members with an event use lipid lowering medication. LDL = low density lipoprotein; HDL = high density lipoprotein.

Autosomal recessive LOF mutations in *KERA* cause cornea plana type 2 (CP2), an ophthalmologic disorder characterised by corneal flattening, but split lamp examination in 2 carriers of the *KERA* p.Ser307Cys mutation did not reveal CP2 characteristics. Additionally, in 9 CP2 cases and their 9 first-degree relatives (age>55 and >65 years for males and females) no evidence of clinical events related with atherosclerosis were detected.

### The Effect of the Mutation on the KERA Protein Structure; *In silico* Analysis (Figure 3)

**Figure 3:**
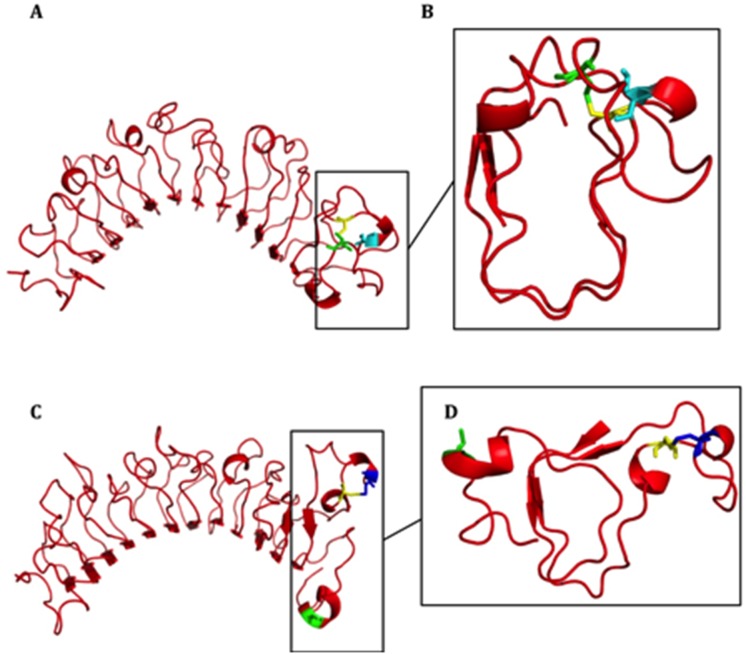
Structures obtained from molecular dynamic simulations of the mutant p.Ser307Cys KERA protein. Molecular dynamic simulations were performed as described in File S1. The residue Cys303 is highlighted in yellow, Cys343 in green, Ser307 in cyan and Cys307 residue in blue. **A**, Structure of wild-type KERA. **C** Structure of the KERA mutant p.Ser307Cys. **B**, A detailed view of the C-terminal part of wild type KERA highlighting the Cys303–Cys343 disulphide bond. **D**, Possible structural effects of the substitution of a serine for a cysteine at residue 307 showing a favourable Cys303–Cys307 disulphide bond. Consequently, Cys343 is available for binding with cysteine residues of other proteins.

The cysteine residues at 303 and 343 in KERA are highly conserved among a large range of animal species. The mutation, which introduces a novel cysteine at residue 307 leads to a substantial change in this conserved region. This was studied in more detail by molecular dynamics (MD) computer simulations started from a homology model based on the crystal structure of decorin. [22] The Ser307Cys mutation is localised in the C-terminal part of KERA and is flanked by cysteine residues at positions 303 and 343, which are assumed to be covalently bonded (Figure 3A). Whilst the horseshoe fold, typical of leucine-rich repeat domain containing proteins, was maintained during simulation, the mutation at residue 307 may form a preferred disulphide bond with Cys303, resulting in Cys343 being unpaired and to become more solvent exposed than in the wild-type KERA protein (Figures 3A–D).

### KERA is Associated with Atherosclerotic Burden in Humans

**Figure 4:**
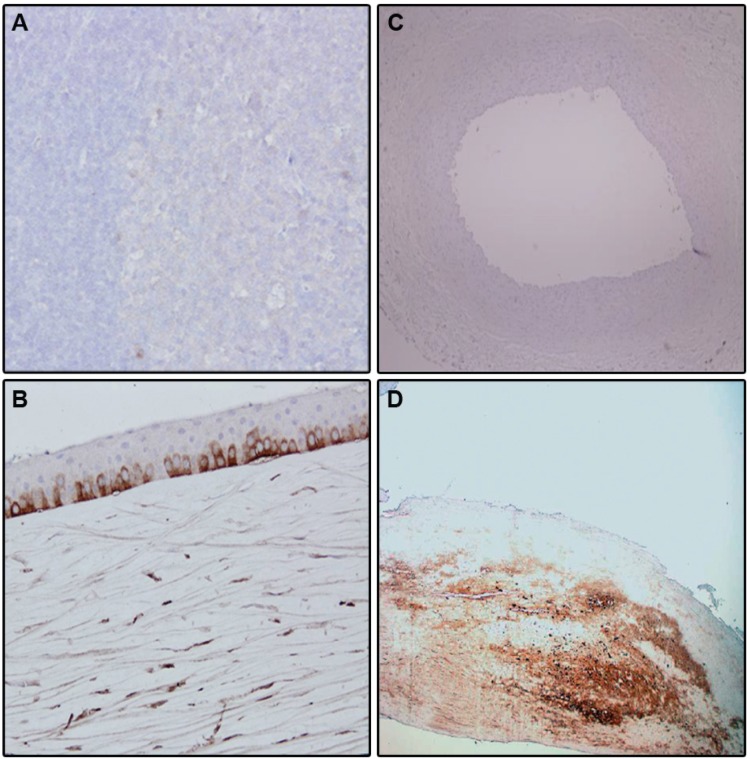
KERA is expressed in atherosclerotic but not in non-diseased arteries. **A**, KERA is not expressed in tonsil tissue; **B**: KERA is expressed in corneal tissue **C**: KERA is not expressed in mammary artery. **D**: KERA is expressed in arterial segments obtained from a patient with the *KERA* mutation.

**Figure 5:**
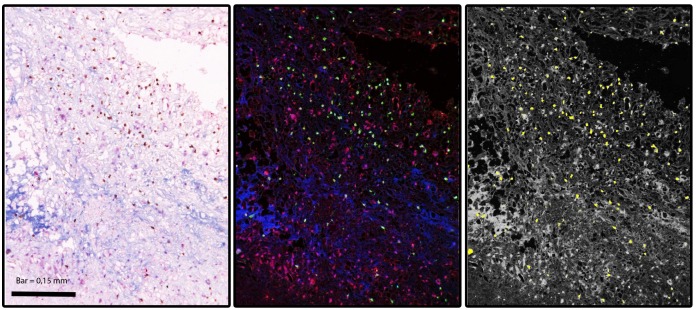
Co-localization of KERA with CXCL1 and CD3 positive type I helper T lymphocytes was assessed in human plaque segments. **A**: triple stain with KERA (blue), CXCL1 (red) and CD3 positive cells (brown). **B**: Spectral Imaging of triple staining. **C**: The yellow staining demonstrates that these cells are positive for all three components.

KERA was not expressed in tonsil tissue (Figure 4A) and mammary artery segments (Figure 4C). KERA was nicely expressed in cornea (Figure 4B). Interestingly KERA was present in a human artery segment obtained from a patient who is a carrier of the *KERA* mutation (Figure 4D). Additionally we have tested arterial segments from 9 patients at different stages of atherosclerosis progression (Figures S2B and S2C in File S1) and found that KERA was not expressed in 2 non-diseased arterial segments (Figure S2A in File S1) but was expressed in atherosclerotic plaque regions. KERA was localised within the extracellular matrix, in close vicinity to the lipid core of the atherosclerotic plaque. Domains rich in KERA showed a strong staining by antibodies against CD3 and Chemokine (C-X-C motif) ligand 1 (CXCL1), indicating the presence of lymphoid T helper (Th1) cells in addition to the myeloid cells (Figures 5A–C).

### KERA Expression is Associated with Atherosclerotic Burden in Apoe^−/−^ Mice

**Figure 6:**
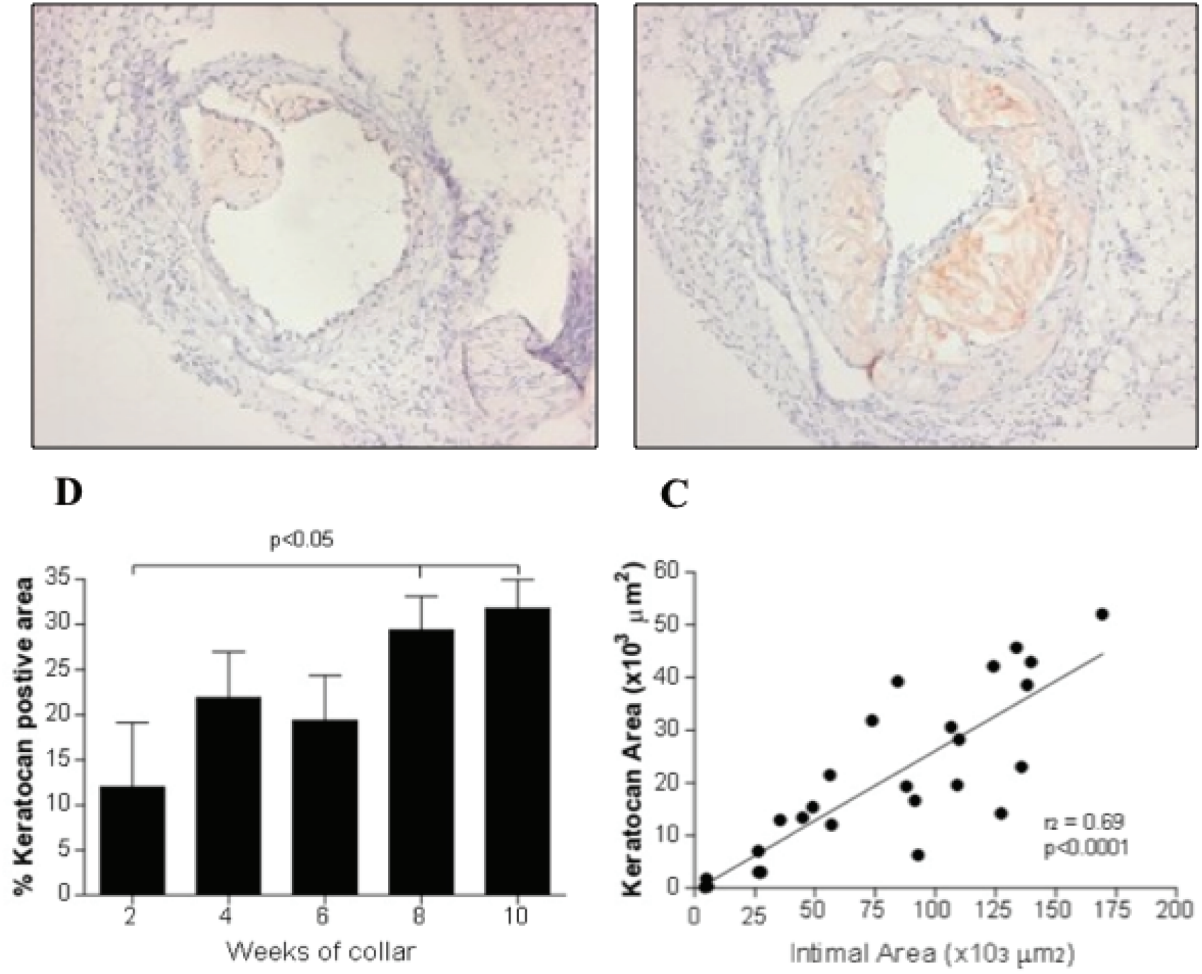
Expression of KERA in atherosclerotic tissue in *Apoe^−/−^* mice after induction of atherosclerosis by collar placement. **A–B**, Early (week = 2, A) and advanced (week = 8, B) atherosclerotic tissue from murine carotid arteries were stained for KERA (brown) and hematoxylin (blue). While present mainly near endothelial cells in early lesion, KERA is predominantly present in the matrix of the plaque at more advanced lesions. **C–D**, KERA expression overtime in *Apoe^−/−^* mice with collar placement show significant correlation with plaque size (r^2^ = 0.69, P<0.0001).

Next we tested whether KERA protein expression was associated with atherosclerotic burden in an established *Apoe^−/−^* mouse model for atherosclerosis, in which atherosclerosis was induced by perivascular collar placement. At different time points after collar placement the degree of atherosclerosis and KERA protein expression was analysed in carotid artery. KERA was detected in both early and advanced atherosclerotic lesions (Figures 6A and B). Interestingly, the extent of atherosclerotic lesion formation, quantified by intima area, was over time significantly correlated with KERA protein expression (r^2^ = 0.69, P<0.0001) (Figures 6C and D).

## Discussion

We identified a novel p.Ser307Cys mutation in the extracellular matrix protein KERA in a large pedigree with a Mendelian form of premature atherosclerosis by linkage analysis combined with next generation sequencing. The variant was absent from nearly 28,000 DNA samples. KERA encodes for a keratan sulfate proteoglycan expressed in the cornea, trachea and at lower levels in the intestine, skeletal muscle, ovary and lung, However no expression in the vasculature has been reported [23]. Interestingly, the protein was absent from healthy artery segments, but heavily abundant within the lipid core of atherosclerotic lesions, which emphasizes that KERA might be a novel actor in the pathobiology underlying atherosclerosis. This notion is further strengthened by the fact that in *Apoe^−/−^* mice, induction of atherosclerosis in the carotid arteries by cuff placements significantly correlated with KERA expression in the plaques.

The mutation introduces a cysteine at residue 307 in the carboxy-terminal of the gene and is flanked by similar residues at positions 303 and 343, which are assumed to be disulphide bonded in wild-type KERA. Molecular dynamic simulations suggested that the new Cys307 favours form a disulphide bond with Cys303, which may lead to improved stability of the protein fold and structure around this carboxy-terminal domain. In addition, during simulation Cys343 was found to be more solvent exposed in the mutant protein thus enabling novel protein-protein interactions. Collectively, the results from the molecular dynamic simulations favour a gain of function (GOF) for the p.Ser307Cys mutation.

The concept of GOF is further substantiated by studies in patients with LOF mutations in KERA. Thus far, autosomal recessive LOF mutations in KERA cause cornea plane type 2 (CP2) (OMIM 121400; 217300), a rare disorder characterized by excessively flat and thin corneas. Worldwide about 100 cases have been described [24]–[28]. An increased risk for atherosclerosis has not been reported in these individuals, but this might have been overlooked because previous studies may have focussed solely on the ophthalmological consequences of *KERA* mutations. However, in the 9 identified CP2 patients in the Netherlands, and in their first degree relatives, no symptoms of premature atherosclerosis were observed, suggesting that both partial or complete LOF for KERA does not confer a substantial increase in the risk of atherosclerosis. No corneal abnormalities were identified in 2 affected family members (IV:I and IV:2, Figure 2B) carrying the novel p.Ser305Cys mutation. The absence of opthalmic effects in the assumed GOF mutation carriers is in line with previous studies, showing that KERA mutations are not identified in subjects suffering from cornea plane type 1 (CP1), the autosomal dominant form of cornea plana [29].

The clinical diversity between heterozygous carriers of our novel GOF *KERA* variant, characterized by atherosclerosis risk, and homozygous carriers of LOF *KERA* variants, resulting in CP2, might be related to the putative GOF effect of the novel mutation. The role of KERA in atherosclerosis has not been investigated so far, but it is interesting to note that KERA has been shown to play a role in neutrophil migration [30]. In mice, neutrophil migration is orchestrated by a chemical gradient of a wide range of chemokines including CXCL1 in the vessel wall [31]. KERA is one of the regulators of the CXCL1 gradient [32]. Interestingly, endothelial CXCL1 has recently been shown to play a crucial role in hyperlipidemia-induced arterial leukocyte arrest [33]. The receptor for CXCL1 CXCR2 is present on myeloid cells like neutrophils and monocyte/macrophages, which are directly involved in all aspects of atherosclerosis [30]. We hypothesize that the GOF mutation observed in the large PAS pedigree results in an augmented binding of KERA to CXCL1, which may lead to increased neutrophil migration into the vessel wall. This notion is supported by recent observations in mice, where both arterial CXCL1 and leukocyte-specific CXCR2 expression are central to macrophage accumulation in established fatty streak lesions [34]. Concomitantly in mice lacking Cxcl1, atherosclerosis is significantly reduced [35]. Notably, in human artery segments we observe a co-localisation of KERA with CXCL1 and the lymphoid T cell marker CD3. Further functional studies are required to confirm our proposed model.

A number of considerations have to be taken into account while interpreting the data. Although the results from this study are suggestive of a role for KERA in atherosclerosis, a direct causative role has not been established thus far.

A specific concern is that neither we, nor others did observe possible *KERA* GOF mutations in other cases with premature atherosclerosis. However, the identification of this extremely rare variant, which is absent from nearly 56,000 alleles and the confirmation of the presence of KERA in the plaque in mice and men does suggest that KERA may be an active player in an, as of yet, not fully elucidated novel pathway in atherosclerosis. Further studies are warranted to confirm our findings and to establish whether KERA might be an attractive target for therapy.

## Supporting Information

File S1 **This file contains a more extended method sextoin is presented including detailed methodologic information and Table S1–Table S4, Figure S1–Figure S2, and a Reference list (References S1).** Table S1, Annotated Genes in linkage interval on chromosome 12. Table S2, SNPs in linkage interval on chromosome 12. Table S3, Demographics of additional relatives of the extensive pedigree. Table S4, Age, BMI and plasma lipids for participants in the PAS cohort (N = 935). Figure S1, Extended pedigree. Figure S2, KERA expression in atherosclerotic plaque segments. References S1.
(DOCX)
