# Supplementary material for: Mutation in KERA Identified by Linkage Analysis and Targeted Resequencing in a Pedigree with Premature Atherosclerosis
Source: PLoS One. 2014 May 30;9(5):e98289. doi: 10.1371/journal.pone.0098289 (PMC4039470; doi:10.1371/journal.pone.0098289)
Supplement: File S1 — This file contains a more extended method sextoin is presented including detailed methodologic information and Table S1–Table S4, Figure S1–Figure S2, and a Reference list (References S1). Table S1, Annotated Genes in linkage interval on chromosome 12. Table S2, SNPs in linkage interval on chromosome 12. Table S3, Demographics of additional relatives of the extensive pedigree. Table S4, Age, BMI and plasma lipids for participants in the PAS cohort (N = 935). Figure S1, Extended pedigree. Figure S2, KERA expression in atherosclerotic plaque segments. References S1. (DOCX) [file pone.0098289.s001.docx]

**Supplementary Data**

**Supplementary Methods**

**Definition of CVD**

CVD was defined by the presence of at least one of the following features: (I) acute myocardial infarction (AMI), proven by at least two of the following criteria: (a) classical symptoms (chest pain that may radiate, oppressive pain, nausea, sweating and absence of chest-wall tenderness on palpation [1]), (b) specific electrocardiographic abnormalities, (c) elevated cardiac enzymes (e. g. troponin [2] and elevated creatine-kinase (CK) and its myocardial band enzyme (CK-MB), levels [3]); (II) percutaneous coronary intervention (PTCA) (III) coronary artery bypass grafting (CABG) (IV) angina pectoris (AP), diagnosed as classical symptoms (recurrent attacks of retrosternal pain brought on by effort and emotion and relieved by rest and the administration of nitroglycerin [4]) in combination with at least one unequivocal result of one of the following: (a) exercise test, (b) nuclear scintigram, (c) dobutamine stress ultrasound, (d) a more than 70% stenosis on a coronary angiogram or (f) requiring treatment (V) ischemic stroke (CVA), demonstrated by CT- or MRI scan. Blood pressure was measured using an oscillometric blood pressure device (Omron 7051T< Hoofddorp, The Netherlands).

**Studies in patients with Cornea Plana 2 and ophthalmologic evaluation on KERA mutation carriers**

Complete loss of function (LOF) variants in *KERA* have been described to cause autosomal recessive Cornea Plana 2 (CP2), a rare disorder characterised by an aberrant large corneal radius curvature, resulting in hypermetropia, astigmatism and poor acuity [5]. Two heterozygous carriers of the novel *KERA* mutation in the family under study (IV:1 and IV:2) underwent split lamp examination to assess whether stigmata of CP2 were present. The Dutch Cornea Physicians Network participated in the study to substantiate the prevalence of premature atherosclerosis in patients with CP2 and their first-degree relatives. Patients were contacted by their treating physicians to obtain data regarding CVD.

**Immunohistochemistry**

*Human plaque*

Coronary arteries were classified according to different stages of atherosclerosis using hematoxylin (Fluka Biochemica, Buchs, Switzerland) and eosin (Sigma Aldrich, St. Louis, MO, USA) stains according to standard protocols. Mammary arteries were included as negative control [6]. Tissue specimen were stained with antibodies against KERA (rabbit anti human KERA clone H-50, Sc-66941, Santa Cruz Biotechnology, Santa Cruz, CA, USA), smooth muscle cell α-actin (SMA) (mouse 1A4 antibody, DAKO, Glostrup, Denmark), macrophage CD68 (mouse monoclonal anti human CD68 PG-M1, DAKO, Heverlee, Belgium), endothelial cell CD34 (mouse anti human CD34Q, Bend10, ThermoFischer Scientific, Waltham, MA, USA), Chemokine (C-X-C motif) ligand 1 (CXCL1) (LS-B2843 antibody, LifeSpan Bioscience, Seattle, WA, USA) and T-cell CD3 (mouse anti-human anti-CD3, IgG monoclonal SP7, ThermoFischer Scientific).

Sections were fi xed in 4% paraformaldehyde and subsequently embedded in paraffin. For immunostaining, paraffin sections were deparaffinised before endogenous peroxidase quenching and heat-induced epitope retrieval (HIER) citrate buffer pH 6.0 (Thermo Scientific). After blocking with Ultra V Block (Thermo Fischer Scientific, Fremont, CA, USA) slides were incubated anti-KERA 1:1000 (rabbit anti human KERA clone H-50, SC-66941, Santa Cruz, USA) overnight at 4ºC. Staining was performed with anti-rabbit Horse Radish Peroxidase (HRP) labelled IgG (ImmunoLogic, Duiven, The Netherlands) followed by Bright DAB^+^ visualization (ImmunoLogic, Duiven, The Netherlands). Counterstaining was performed using hematoxylin and slides were cover-slipped with VectaMount (Vector Laboratories, Burlingname, CA, USA). Positive controls consisted of samples of human cornea and human tonsil. Negative controls consisted of experimental tissues stained without the addition of primary antibodies following the same protocol. Microscopy pictures were analysed by Adobe Photoshop CS4.

To explore cellular localization of KERA in human atherosclerotic lesions the sequential alkaline phosphatase (AP) double staining method was used as described elsewhere [7]. KERA was stained in combination with either smooth muscle cell α-actin (SMA) (mouse 1A4 antibody, DAKO, Glostrup, Denmark), macrophage CD68 (mouse monoclonal anti human CD68 PG-M1, DAKO, Heverlee, Belgium) or endothelial cell CD34 (mouse anti human CD34Q, Bend10, ThermoFischer Scientific, Waltham, MA, USA). Visualization was performed with vector blue (Vector Laboratories) for KERA and vector red (Vector Laboratories) for CD68, CD34 and SMA. Co-localization of KERA, T-cells CD3 and Chemokine (C-X-C motif) ligand 1 (CXCL1) was performed in human atherosclerotic lesions by triple staining with rabbit anti-human CXCL1 (1:500; LS-B2843 antibody, LifeSpan Biosciences, Seattle. WA, USA) and rabbit anti human CD3 (1:5000;^,^ IgG monoclonal SP7, ThermoFisher Scientific). After overnight incubation with KERA antibody the tissue slides were incubated with anti-rabbit HRP labelled IgG (ImmunoLogic) and visualized with Dyoblue 1:100 (Dyomics, Jena, Germany). Subsequently, a protein block with Ultra V Block was performed, followed by incubation with the CXCL1 antibody for 60 min, followed by incubation with anti-rabbit HRP labelled IgG and visualization with vector red (Vector Laboratories). Then antigen retrieval in HIER citrate buffer pH 6.0, was done, followed by overnight incubation with CD3 antibody. Finally, for visualization the tissue slides were incubated with anti-rabbit HRP labelled IgG followed by addition of DAB^+^ and cover slipped. Immuno-triple stained tissue slides were analysed using Nuance 3.0 Spectral Imaging (Caliper Life Sciences, Hopkinton, MA, USA), a computer assisted optical technique to get unblended colours in the section based on their spectral characteristics. This allows detailed analysis of the triple stained cells as described elsewhere [8].

*Mice plaque assessment*

Cryostat sections of the aorta root (n = 7; 10 mm) were embedded in Tissue Tek (Sakura Finetek, Alphen a/d Rijn, The Netherlands). Tissue slides were washed in phosphate buffered saline (PBS) and blocked with 5% milk powder, followed by incubation with KERA 1:1000 (clone H-50, SC-66941). As secondary antibody anti-rabbit HRP labelled IgG was used and visualization with vector red was performed. The slides were counterstained with hematoxyline.

Microscopy pictures were analysed using Leica Owin Image analysis software (Leica Microsystems, Rijswijk).

**Molecular Dynamics Modeling Simulations**

To investigate the possible effects of the p.Ser307Cys mutation on the structure of KERA, molecular dynamics (MD) computer simulations were performed. A homology model based on s crystal structure of Decorin (PDB code: 2FT3) [9], a protein showing great homology to KERA, was obtained from the SWISS-Model repository (model date: 2012-09-21) [10,11]. Energy minimization and simulations were performed using the GROMOS11 biomolecular simulation software [12,13] in combination with the GROMOS 54A7 force-field parameter set to describe the protein and counter ions [14].

In a ﬁrst step, the (steepest-descent) energy-minimized KERA structure was solvated in a cubic periodic box with lengths of 10.0 nm, containing approximately 30 000 SPC molecules [15], after which 40 chlorine ions were added to neutralize the net charge of the system. The system was heated up to 300 K (in ﬁve steps of 60 K) in NVT simulations of 20 ps each, in which protein atoms were restrained to their energy-minimized position using gradually decreasing force constants. Subsequently, a data production simulation of 10 ns at 300 K was performed, in which structural data were stored every 2 ps.

During MD a time step of 2 fs was used, and the volume of the system was kept constant at 1000 nm^3^. Newton’s equations of motion were integrated using a Leap-frog algorithm [16]. Bond lengths were constrained using the SHAKE algorithm with a relative geometric tolerance of 10^−4^ [17]. The temperature of the protein and solvent were maintained at their reference values by coupling to two separate Berendsen thermostats [18], both with a coupling time of 0.1 ps. Non-bonded interactions were treated using a triple-range cut off: interactions within 0.8 nm were evaluated at every time step, and interactions at a distance between 0.8 and 1.4 nm were evaluated every ﬁfth step, together with the update of the pairlist. Long-range electrostatic interactions were represented by a reaction ﬁeld [19], using a dielectric constant of 61.

Additional MD simulations were performed of wild-type KERA in which the C303-C343 sulphur bridge was removed (WTnS), of S307C KERA in which no sulphur bridges were present between C-terminal residues C303, C307 and C343 (MUTnS), and of S307C KERA with a C303-C307 sulphur bridge (MUT). The starting coordinates of WTnS were identical to the starting coordinates of WT. The MUTnS production simulation started from an equilibrated WTnS coordinate set (taken after 2 ns of WTnS simulation), in which the S307C mutation was manually introduced using the MOE software package [20]. The starting structure of the MUT simulation was obtained by introducing the C303-C307 sulphur bridge into a coordinate set taken from the MUTnS, in which the distance between the C303 and C307 sulphur atoms was minimal (0.30 nm). Subsequent energy minimization, thermal equilibrations and 10 ns production simulations were performed according to the protocol described for WT.

**Table S1**

Annotated Genes in linkage interval on chromosome 12

| Gene | Name | location |
| --- | --- | --- |
| DUSP6  GALNT4  POC1B  ATP2B1  CCER1  EPYC  KERA  LUM  DCN  BTG1  CLLU1OS  CLLU1  PLEKHG7  EEA1  NUDT4  UBE2N  MRPL42  SOCS2  CRADD | Dual Specificity Phosphatase 6  UDP-N-acetylgalactosaminyltransferase 4  POC1 centriolat protein homolog B  ATPase, C++ transporting plasma membrane 1  Coiled-coil glutamate-rich protein 1  Epiphycan 1  Keratocan  Lumican  Decorin  B-cell translocation 1  Chronic lymphocytic leukemia up-regulated 1 opposite strand  Chronic lymphocytic leukemia up-regulated 1  Plecktstring homology domain containing family G 7  Early ensosome antigen 1  Nudix-type motif 4  Ubiquitin-conjugating enzyme E2N  Mitochondrial ribosomal protein L42  Suppressor of cytokine signalling 2  CASP2 and RIPK1 domain containing adaptor with death domain | 89,754,816  89,920,535  89,933,039  89,979,826  91,343,992  91,355,456  91,442,268  91,495,232  91,537,035  92,532,054  92,811,870  92,815,735  93,128,265  93,164,285  93,769,701  93,800,088  93,859,270  93,961,598  94,069,151 |

**Table S2.** SNPs in linkage interval on chromosome 12

| SNP | LOD score |
| --- | --- |
| rs1688545  rs796051  rs6538188  rs12817819  rs11105382  rs17783015  rs2722222  rs2553101  rs10777205  rs2553110  rs11105458  rs4842697  rs7306228  rs10777230  rs2579088  rs1438993  rs7139199  rs11105554  rs1347846  rs17836972  rs1470032  rs12818395  rs6538233  rs826210  rs1222513  rs1368013  rs2116497  rs704106  rs7960740  rs1148679  rs1432113  rs2188546  rs2723893  rs2723902  rs2520500  rs763564  rs11503340  rs10859024  rs1604819  rs1948839  rs7954238  rs1354421  rs9888417  rs4842732  rs1847458  rs10859057  rs4272843  rs7313740  rs7358706  rs17018653  rs10777287  rs3759222  rs704144  rs973871  rs1125888  rs4842763  rs11106135  rs7980632  rs10745558  rs4399376  rs11106219  rs11106224  rs11612413  rs10859177  rs6538290  rs7294593  rs7960107  rs4240750  rs10859227  rs4842814  rs4567507  rs1979705  rs12312031  rs6538319  rs709240  rs1836681  rs1190655  rs1798059  rs790455  rs961810  rs7980688  rs11106496  rs453629  rs1086584  rs337655  rs7972305  rs1515560  rs7298571  rs9971898  rs12425333  rs10777409  rs7980716  rs1030810  rs10777428  rs7976370  rs7304800  rs11106676  rs17194402  rs4760506  rs12319590  rs11609317  rs7296486  rs11830779  rs10859402  rs4760420  rs7956439  rs4761716  rs10777477  rs11106945  rs10859481  rs10859482  rs7976742  rs7975732  rs3867978  rs10859513  rs10859522  rs7488123  rs11611927  rs1493848 | -3,85  2,22  2,88  3,23  3,31  3,31  3,31  3,31  3,31  3,31  3,31  3,31  3,31  3,31  3,31  3,31  3,31  3,31  3,31  3,31  3,31  3,31  3,31  3,31  3,31  3,31  3,31  3,31  3,31  3,31  3,31  3,31  3,31  3,31  3,31  3,31  3,31  3,31  3,31  3,31  3,31  3,31  3,31  3,31  3,31  3,31  3,31  3,31  3,31  3,31  3,31  3,31  3,31  3,31  3,31  3,31  3,31  3,31  3,31  3,31  3,31  3,31  3,31  3,31  3,31  3,31  3,31  3,31  3,31  3,31  3,31  3,31  3,31  3,31  3,31  3,31  3,31  3,31  3,31  3,31  3,31  3,31  3,31  3,31  3,31  3,31  3,31  3,31  3,31  3,31  3,31  3,31  3,31  3,31  3,31  3,31  3,31  3,31  3,31  3,31  3,31  3,31  3,31  3,31  3,31  3,31  3,31  3,31  3,31  3,31  3,31  3,31  3,31  3,31  3,31  3,31  3,30  3,24  -2,29 |

**Table S3:** Demographics of additional relatives of the extensive pedigree.

|  | *KERA* wild-type carrier  (n = 2) | *KERA* Mutant carrier  (n = 10) |
| --- | --- | --- |
| Age | 60/70 | 57.6 ± 11.6 |
| Sex (male/female) | 1/1 | 7/3 |
| BMI (kg/m^2^) | 26/33 | 32 ± 6 |
| Total cholesterol (mmol/l) | 7.2/4.3 | 5.3 [3.8 – 6.0] |
| low-density lipoprotein cholesterol (mmol/l) | 5.2/2.4 | 3.2 [2.2 – 4.3] |
| high-density lipoprotein cholesterol (mmol/l) | 1.3/0.9 | 1.2 [1.1 – 1.3] |
| Triglyceride (mmol/l) | 1.4/2.2 | 1.3 [1.0 – 1.8] |

Data are expressed as mean ± standard deviation. Lipid parameters are expressed as median with interquartile range (IQR). Pedigree members with an event use lipid lowering medication (see supplemental Figure 1). LDL = low density lipoprotein; HDL = high density lipoprotein.

**Table S4.** Age, BMI and plasma lipids for participants in the PAS cohort (N = 935).

| **Male/Female** | **Age (years)** | **BMI** | **LDL-c** | **HDL-c** | **TG** |
| --- | --- | --- | --- | --- | --- |
| 708/227 | 43 ± 5 | 26.9 ± 1.2 | 3.11 ± 1.29 | 1.14 ± 0.32 | 2.06 ± 3.41 |

Data are expressed as number (N) and presented as mean ± standard deviation. BMI = body mass index in kg/m^2^; LDL-c = low-density lipoprotein cholesterol in mmol/l; HDL-c = high-density lipoprotein cholesterol in mmol/l; TG = plasma triglycerides in mmol/l.

**Figure S1:** Extended pedigree

The extended pedigree contains members that have been tested for the *KERA* variant (p.Ser305Cys, c.920C>G) including the core pedigree. In the pedigree we show carriership for the *KERA* mutation for each relative and the type of event and the age at which the event occurred. The arrow indicates the index case. *These two female individuals, aged 51 and 56 years, are still below the PAS age (<65 years), yet we have no clinical data available to mark their affection status. One brother of the index aged 73 has no signs of atherosclerotic disease. AMI= acute myocardial infarction; TIA = transient ischemic attack; PTCA = percutaneous transluminal coronary angioplasty; CVA = cerebrovascular accident; AP = angina pectoris; CABG = coronary artery bypass graft; ACS = acute coronary syndrome; imaging evidence is defined as a calcium score above 90^th^ percentile for age and gender on coronary CT.

**
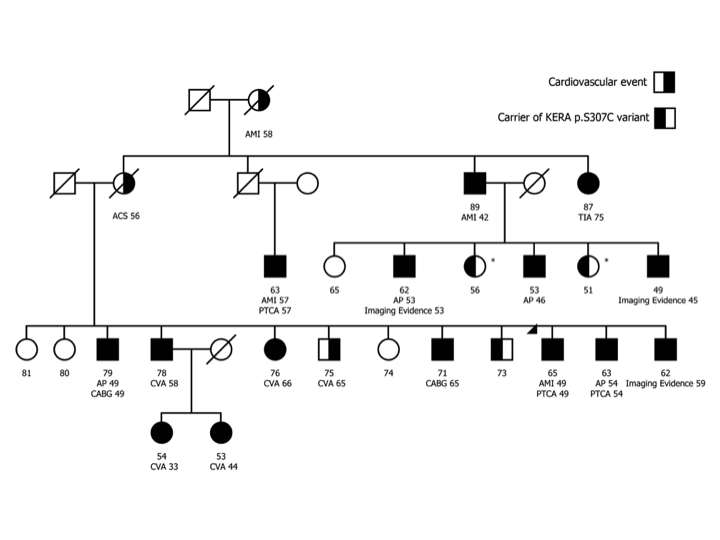
**

**Figure S2:** KERA expression in atherosclerotic plaque segments.

**A:** KERA is not expressed in a healthy arterial segment. **B and C:** KERA (Blue) and CD68 (red) are present in the same plaque area in an early lesion (B) and in a late lesion (C).

**
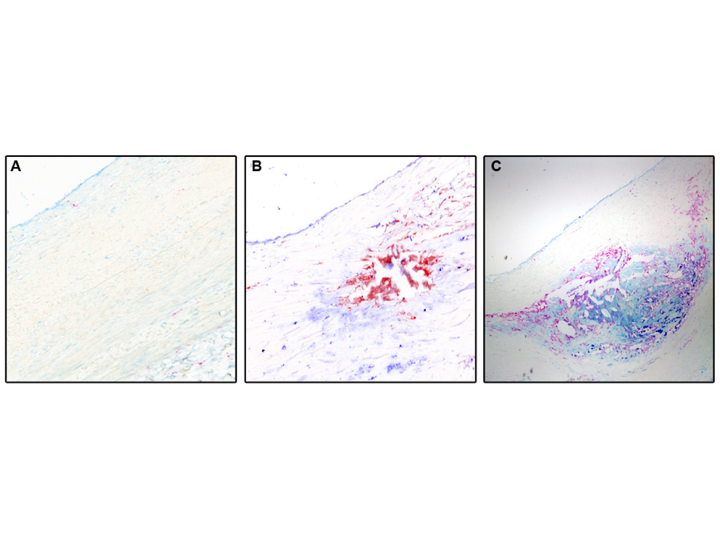
**

**Supplementary reference list**

1. Bruyninckx R, Aertgeerts B, Bruyninckx P, Buntinx F (2008) Signs and symptoms in diagnosing acute myocardial infarction and acute coronary syndrome: a diagnostic meta-analysis. Br J Gen Pract 58: 105-111.

2. Antman EM, Tanasijevic MJ, Thompson B, Schactman M, McCabe CH et al. (1996) Cardiac-specific troponin I levels to predict the risk of mortality in patients with acute coronary syndromes. N Engl J Med 335: 1342-1349.

3. Myocardial infarction redefined--a consensus document of The Joint European Society of Cardiology/American College of Cardiology Committee for the redefinition of myocardial infarction (2000) Eur Heart J 21: 1502-1513.

4. Scherf D, Cohen J (1974) [Editorial: "Variant" angina pectoris]. Circulation 49: 787-789.

5. Ebenezer ND, Patel CB, Hariprasad SM, Chen LL, Patel RJ et al. (2005) Clinical and molecular characterization of a family with autosomal recessive cornea plana. Arch Ophthalmol 123: 1248-1253.

6. de Boer OJ, van der Meer JJ, Teeling P, van der Loos CM, van der Wal AC (2007) Low numbers of FOXP3 positive regulatory T cells are present in all developmental stages of human atherosclerotic lesions. PLoS One 2: e779.

7. van der Loos CM (2008) Multiple immunoenzyme staining: methods and visualizations for the observation with spectral imaging. J Histochem Cytochem 56: 313-328.

8. Meijer-Jorna LB, Aronica E, van der Loos CM, Troost D, van der Wal AC (2012) Congenital vascular malformations--cerebral lesions differ from extracranial lesions by their immune expression of the glucose transporter protein GLUT1. Clin Neuropathol 31: 135-141.

9. Scott PG, Dodd CM, Bergmann EM, Sheehan JK, Bishop PN (2006) Crystal structure of the biglycan dimer and evidence that dimerization is essential for folding and stability of class I small leucine-rich repeat proteoglycans. J Biol Chem 281: 13324-13332.

10. Kiefer F, Arnold K, Kunzli M, Bordoli L, Schwede T (2009) The SWISS-MODEL Repository and associated resources. Nucleic Acids Res 37: D387-D392.

11. Kopp J, Schwede T (2004) The SWISS-MODEL Repository of annotated three-dimensional protein structure homology models. Nucl Acids Res 32: D230-D234.

12. Kunz AP, Allison JR, Geerke DP, Horta BA, Hunenberger PH et al. (2012) New functionalities in the GROMOS biomolecular simulation software. J Comput Chem 33: 340-353.

13. Schmid N, Allison JR, Dolenc J, Eichenberger AP, Kunz AP et al. (2011) Biomolecular structure refinement using the GROMOS simulation software. J Biomol NMR 51: 265-281.

14. Schmid N, Eichenberger AP, Choutko A, Riniker S, Winger M et al. (2011) Definition and testing of the GROMOS force-field versions 54A7 and 54B7. Eur Biophys J 40: 843-856.

15 Berendsen HJC, Postma JPM, van Gunsteren, Hermans J (1981) Intermolecular forces. Reidel, Dordrecht, The Netherlands.

16 Hockney RW (1970). The potential calculation and some applications. Methods Comput Phys 9:136–211.

17 Ryckaert JP, Ciccotti G, Berendsen HJC (1977) Numerical integration of the cartesian equations of motion of a system with constraints: Molecular dynamics of n-alkanes. J Comput Phys 23:327–34.

18 Berendsen HJC, Postma JPM, van Gunsteren WF, ADiNola A, Haak JR (1984) Molecular-dynamics with coupling to an external bath. J Chem Phys 81:3684–3690.

19 Tironi IG, Sperb R, Smith PE, van Gunsteren WF (1995) A generalized reaction ﬁeld method for molecular dynamics simulations. J Chem Phys 102:5451–5495.

20 Molecular Operating Environment (MOE) (2012) 10 Chemical Computing Group Inc., 1010 Sherbooke St. West, Suite 910, Montreal, QC, Canada, H3A 2R7.
